# Tau reduction in aged mice does not impact Microangiopathy

**DOI:** 10.1186/s40478-020-01014-4

**Published:** 2020-08-18

**Authors:** Rachel E. Bennett, Miwei Hu, Analiese Fernandes, Marta Perez-Rando, Ashley Robbins, Tarun Kamath, Simon Dujardin, Bradley T. Hyman

**Affiliations:** Department of Neurology, MassGeneral Institute for Neurodegenerative Disease, Massachusetts General Hospital, Harvard Medical School, Charlestown, MA 02129 USA

**Keywords:** Alzheimer’s disease, Blood vessels, Brain microvessels, Tau, Microangiopathy, Neurodegeneration

## Abstract

Microangiopathy, including proliferation of small diameter capillaries, increasing vessel tortuosity, and increased capillary blockage by leukocytes, was previously observed in the aged rTg4510 mouse model. Similar gene expression changes related to angiogenesis were observed in both rTg4510 and Alzheimer’s disease (AD). It is uncertain if tau is directly responsible for these vascular changes by interacting directly with microvessels, and/or if it contributes indirectly via neurodegeneration and concurrent neuronal loss and inflammation. To better understand the nature of tau-related microangiopathy in human AD and in tau mice, we isolated capillaries and observed that bioactive soluble tau protein could be readily detected in association with vasculature. To examine whether this soluble tau is directly responsible for the microangiopathic changes, we made use of the tetracycline-repressible gene expression cassette in the rTg4510 mouse model and measured vascular pathology following tau reduction. These data suggest that reduction of tau is insufficient to alter established microvascular complications including morphological alterations, enhanced expression of inflammatory genes involved in leukocyte adherence, and blood brain barrier compromise. These data imply that 1) soluble bioactive tau surprisingly accumulates at the blood brain barrier in human brain and in mouse models, and 2) the morphological and molecular phenotype of microvascular disturbance does not resolve with reduction of whole brain soluble tau. Additional consideration of vascular-directed therapies and strategies that target tau in the vascular space may be required to restore normal function in neurodegenerative disease.

## Introduction

Vascular dysfunction is increasingly recognized as a co-occurring pathological feature of neurodegenerative diseases including Alzheimer’s [3, 20]. The Alzheimer’s Disease Neuroimaging Initiative identified that MRI measures of cerebral blood flow were among the first alterations in human subjects with mild cognitive impairment [36], and several other groups have confirmed disturbed blood flow with increasing clinical disease [15, 25, 48, 75]. Reductions in blood flow and other vascular pathologies evident by neuroimaging have been shown to be closely related to the neuropathological accumulation of tau protein [16, 40]. In tauopathies including Alzheimer’s disease and frontotemporal lobar dementia with *MAPT* mutations, regional changes in cerebral blood flow have been observed [10, 24, 64], implicating vascular dysfunction as a common feature of diseases that include tau accumulation.

Two broad explanations for these relationships were considered: altered tissue metabolism in the presence of neurodegeneration due to neuronal loss and diminished metabolic demand [50], or processes related to inflammation [28] arising from a direct action of tau on the microvasculature. Pathological accumulation of Alzheimer’s disease proteins are known to have consequences on vasculature in the brain. Both oligomeric and deposited amyloid beta, for example, are toxic to vascular cells in penetrating arterioles, accumulating within smooth muscle and endothelial cells over time and contributing to vascular dysfunction and cell death in cerebral amyloid angiopathy (CAA) [1, 6, 13, 38, 41, 68, 69]. In addition to CAA, mice that develop amyloid beta plaques also have reduced cerebral blood flow, altered vascular density, and abnormal vascular morphology [11, 22, 45].

More recently, the release of tau by neurons into the extracellular space and its subsequent uptake into non-neuronal cells has been highlighted as a major consideration for disease pathophysiology [32, 43, 51, 60]. Of note, tau protein accumulation in and around vascular cells has been reported and may provide a direct link to vascular dysfunction [18, 44, 53, 72]. Exploring this possibility, animal studies have uncovered a role for tau in altering blood vessel function. In the rTg4510 P301L tau overexpressing mouse model, disrupted blood flow was evident by increased numbers of capillaries occluded by leukocytes [8]. This pronounced phenotype was accompanied by increased numbers of small diameter capillaries (< 5 μm), increased vessel tortuosity and upregulated endothelial cell expression of angiogenesis and hypoxia-related genes [8].

To better understand the relationship between tau protein accumulation and vascular alterations, we made use of the tetracycline-repressible promoter in the rTg4510 mouse model to determine if turning off tau expression impacts the microangiopathy phenotype that was observed previously. These data have important implications for tau-reduction based therapies that are currently in development [21] and suggest that depending on the timing of events, alterations in vascular function may not be impacted by tau suppression.

## Methods

### Animals

Mice used in these experiments were handled and housed according to the animal protocols of Massachusetts General Hospital and the McLaughlin Research Institute. The rTg4510 mice (FVB-Tg4510xB6.TgCK-tTA) are commercially available from Jackson Laboratories (stock no. 024854) and have been extensively characterized [26, 56, 61, 63]. Wild-type littermates included in these studies possessed either the tetO-MAPT*P301L or the Camk2a-tTA transgenes. At 12 months of age, equal numbers of male and female mice were randomized to experimental groups and fed chow containing doxycycline (*n* = 6 wild-type and *n* = 6 rTg4510, Bio-Serv cat no. S3888, 200 mg/kg) or standard chow (*n* = 6 wild-type and *n* = 6 rTg4510, Lab Diet cat no. RMH3000 5P75). A second group of male transgenic mice was treated identically and used for blood vessel isolations (*n* = 4 standard chow, *n* = 3 doxycycline chow). Doxycycline results in repression of the tau transgene in these mice [61].

### In vivo imaging

Following a 12-week treatment, all mice were anesthetized with ketamine/xylazine and underwent a cranial window procedure to remove the skull overlying the somatosensory cortex and a glass coverslip was secured with dental cement. Fluorescein-conjugated 70 kDA dextran was injected intravenously (100 μl, 12.5 mg/ml, Invitrogen) and vasculature was immediately imaged using an Olympus two-photon microscope equipped with a Ti: Sapphire laser (MaiTai, Spectra Physics, 800 nm excitation). A minimum of six regions of interest containing vessels < 20 μm in diameter (capillaries) were imaged with a 25x (n.a. = 1.05) water immersion lens at 3x digital zoom. Z-stacks began 50 μm below the surface of the cortex and included 50 planes at 2-μm steps for a total imaging depth of 100 μm.

Mice were immediately killed by isofluorane and cardiac puncture following imaging. One hemisphere was fixed for histology and one hemisphere was dounce homogenized in phosphate buffered saline (PBS) with protease inhibitors (Roche) and centrifuged at 3000 x g for 10 min. The resulting supernatant was reserved for HEK cell assay, western blotting, and ELISA. The pellet was reserved for subsequent sarkosyl extraction.

For analysis of images, Analyze 3D objects in Image J was used to quantify blood vessel volumes and the Skeletonize function was used to measure lengths. Cortical thickness measurements from histological slices were used to scale these values and take into account atrophy in the rTg4510 mouse model. For quantitative measurements of the number of vessels without blood flow (blocked capillaries), the number of unique blood vessel branches were manually counted in Image J and the presence of red blood cells (which appear black within fluorescently labeled lumens) was tracked. If a red blood cell appeared in the same location of a capillary in at least two planes of the z-stack, the vessel was considered blocked.

### Human tissue selection

Human tissue was provided by the Massachusetts Alzheimer’s Disease Research Center, which conducted an extensive neuropathological characterization of each case. Tissue was selected based on the presence or absence of tau pathology and neuropathological diagnosis [33]. A total of *n* = 9 cases with Alzheimer’s disease pathology (Braak stage V-VI) and *n* = 9 control cases (Braak stage 0-II) were used for microvessel isolation from temporal cortex (Brodmann area 22). Both groups included five male and four female subjects of similar ages (average 83 +/− 9 STD and 80 +/− 15 years old respectively). Tissue was specifically selected from these Alzheimer’s subjects because they lacked concurrent cerebral amyloid angiopathy (CAA), though evidence of cerebrovascular disease was noted in three cases. One control case showed evidence of focal mild CAA in leptomeningeal vessels.

To compare another tauopathy, frontotemporal lobar dementia (FTLD) with tau pathology cases were selected (*n* = 5) and corresponding control cases (*n* = 5) were similarly sex matched though the control group was on average greater in age than the FTLD group (93 +/− 5 vs. 73+/− 15 years). Frontal cortex (Brodmann area 9) was used. Two of these FTLD cases were mutant *MAPT(P301L)* mutation carriers and the other three had no known familial FTLD mutations. All FTLD-tau cases had evidence of tau accumulation in neurons including dystrophic neurites, neurofibrillary tangles, and Pick bodies and did not have TDP-43 pathology. One familial FTLD-tau carrier also had amyloid beta deposition in cortex and hippocampus consistent with Thal stage 2 and one had amyloid beta deposition consistent with Thal stage 1. Additional details of all human tissue can be found in Supplemental Table 1.

From each frozen tissue block, ~ 250 mg of grey matter was separated and used for microvessel isolation and additional tissue (when available) was dounce homogenized in PBS with protease inhibitors, centrifuged at 3000 x g for 10 min to remove debris, and reserved for HEK cell assay, western blotting, and ELISA.

### Microvessel isolation and Immunofluorescent labeling

Blood vessels were isolated from frozen mouse and human tissue following published protocols [14] with modifications. First 200–300 mg tissue was minced into small ~ 1–2 mm pieces with a razor blade in ice cold buffer (Hanks Balanced Salt Solution with 10 mM HEPES, pH 7; ThermoFisher Scientific) and then manually dounce homogenized with 12 up/down strokes. The resulting homogenate was transferred to a 50 mL conical containing 20 mLs buffer and centrifuged at 2000 x g for 10 min at 4C. The supernatant was discarded and the pellet was resuspended by shaking in 20 mls of myelin removal buffer (18% dextran in HEPES-Buffered HBSS). Samples were centrifuged at 4400 x g for 15 min at 4C. The myelin layer was removed by carefully pouring out the supernatant. The pellet was resuspended in 2 mL chilled 1% bovine serum albumin (BSA; Sigma-Aldrich) HEPES-Buffered HBSS and filtered through a 20 μm mesh (Millipore). The filter was rinsed with 30 mLs 1% BSA HEPES-Buffered HBSS and then blood vessels were collected by immersing the filter in a new conical containing 30 mLs of the same buffer and centrifuging for 5 min at 2000 x g at 4 C.

For western blotting, the resulting pellets were then rinsed in PBS (pH 7) twice to remove BSA and then sonicated in PBS with proteinase inhibitors. For immunofluorescent labeling, pellets were fixed in 4% PFA, rinsed in PBS, and then blocked in 5% goat serum 0.25% Triton-X in PBS before incubating overnight in primary antibodies. All antibodies were used at a 1:200 dilution including: mouse anti-ZO-1 (ThermoFisher, cat no. 33–9100, RRID: AB_87181), rabbit anti-Collagen IV (Bio-Rad, cat no. 2150–1470, RRID:AB_2082660), mouse anti-smooth muscle actin (Sigma-Aldrich, cat no. A5228, RRID: AB_262054), rabbit anti-GLUT1 (Millipore, cat no. 07–1407, RRID: RRID:AB_10616217), or mouse anti-tau (ThermoFisher, cat no. MN1000B, RRID: AB_223453). Alexafluor-conjugated anti-mouse or anti-rabbit secondaries were incubated at 1:1000 in blocking buffer the following day. After the final wash in PBS, vessels were resuspended in 10 μl of Fluoromount-G mounting media with DAPI (Southern Biotech) and imaged using a confocal microscope (Olympus FV3000).

### Tau bioactivity assay

Human embryonic kidney cells (HEK-293) stably expressing a CFP/YFP FRET biosensor containing the tau repeat domain (ATCC, cat no. CRL-3275) were cultured in 96-well plates to ~ 70% confluency and used to assess tau seeding bioactivity following published protocols [31]. Protein extracts were prepared from either total cortex, blood vessels, or whole blood (obtained from rTg4510 facial vein draw) and were diluted to a total protein concentration of 1 mg/mL in PBS. One microgram of protein in 1% lipofectamine OPTIMEM was added to triplicate wells and incubated with cells for 24 h. Cells were detached using 1X trypsin, fixed in 2% paraformaldehyde, rinsed in PBS, and flow cytometry measures of the percent of cells containing aggregates and the median fluorescence intensity (ex. 405 nm, em. 525 nm). The percentage of aggregate containing cells and median fluorescence intensity was multiplied to give the integrated FRET density (IFD), reported here as tau bioactivity arbitrary units. All measures were normalized to a lipofectamine only (FRET negative) control.

### Sarkosyl insolubility assay

Tissue pellets were subject to biochemical fractionation to measure soluble and insoluble tau species following previously published protocols [23]. In short, pellets are homogenized in extraction buffer (20 mM Tris-HCl pH 7.5 with 1 mM Dithiothreitol, 1 mM Egtaizic Acid) containing 1% Triton-X and incubated for 30 min at 37 °C before centrifuging at 100,000 x g for 30 min at 4 °C. The supernatant was used as the Triton-X fraction and the pellet was resuspended in extraction buffer containing 1% sarkosyl. After incubation for 30 min at 37 °C, samples were centrifuged at 100,000 x g for 30 min at 4 °C and the sarkosyl soluble supernatant was reserved. Sarkosyl insoluble proteins were solubilized by briefly sonicating in 8 M urea in 50 mM Tris-HCl pH 7.5. Protein concentrations of all fractions were measured by BCA.

### Western blotting

Samples were prepared for Western blots using NuPAGE LDS Sample Buffer and Sample Reducing Agent (Invitrogen). Ten micrograms of protein per lane was loaded on a NuPAGE 4–12% Bis-Tris gel and run at 120 V for 90 min in MES SDS Running Buffer (Invitrogen) before transferring to 0.2 μm nitrocellulose (Invitrogen) at 90 V for 90 min. Blots were rinsed in Tris-buffered saline (TBS) and blocked in Odyssey Blocking Buffer (LI-COR) prior to incubation with primary antibodies. Antibodies that were used in these experiments include: rabbit anti-Tau (DAKO, cat no. A0024, RRID: AB_10013724), mouse anti-human tau (HT7, Invitrogen, cat no. MN1000, RRID: AB_2314654), mouse anti-tau (Tau46, Cell Signaling Technology, cat no. 4019S, RRID:AB_10695394), mouse anti-NeuN (Millipore, cat no. MAB377, RRID: AB_2298772), rabbit anti-Glut1, and chicken anti-Tubulin (Aves Labs, cat no. TUJ, RRID: AB_2315518). For protein loading normalization of sarkosyl fraction experiments, prior to incubation with antibodies, blots were rinsed in distilled water and incubated with Revert 700 Total Protein Stain (LI-COR) following the manufacturer’s instructions.

### Histology

One hemisphere from each mouse brain was drop fixed in 4% paraformaldehyde for 48 h and then equilibrated in 30% sucrose before sectioning on a freezing microtome. Nine sets of 40 μm thick sections were collected such that each set contained sections at 360 μm intervals. For immunostaining, sections were rinsed in TBS and endogenous peroxidases were blocked with 0.3% H2O2 in TBS for 10 min and then incubated in blocking buffer containing 3% normal goat serum in TBS with 0.25% Triton-X (TBS-X) for 30 min. Anti-Tau (ThermoFisher, cat no. MN1000B) was applied overnight at 4C in blocking buffer and then detected with 1:200 ABC horse-radish peroxidase (Vector Labs, cat no. PK-6100) and diaminobenzidine (DAB, Sigma-Aldrich). For tau detection, the biotinylated antibody was used to avoid non-specific mouse IgG labeling. Alternatively, horse anti-mouse IgG was incubated for 3 h at room temperature in 2% normal horse serum in TBS-X before following the same HRP detection procedure (Vector labs, cat no. PK-4002).

For immunofluorescent labeling, sections were rinsed in TBS, incubated in 3% NGS TBS-X for 30 min and then incubated overnight in blocking buffer with anti-human tau (biotinylated HT7 to avoid cross-reactivity with endogenous mouse IgG) and anti-Glut1. Alexa-fluor 555-conjugated streptavidin and alexa-fluor 488-conjugated anti-rabbit IgG were applied in TBS-X the following day and images were captured using an Olympus VS-120 slide scanning system with a 20x objective.

For in situ hybridization, a human *Mapt* probe that does not cross-react with mouse tau was purchased from Advanced Cell Diagnostics (cat no. 417491). In situ hybridization was performed according to the manufacturer’s instructions using the RNAscope Multiplex Fluorescent V2 Assay (Advanced Cell Diagnostics, cat no. 23110). Glut1 antibody labeling and subsequent imaging of in situ fluorescence (Cy5 labeled) and Glut1 (Alexa 488 labeled) was performed using an Olympus FV3000RS confocal microscope.

### Quantitative PCR on mouse brains

RNA was isolated from 20 to 30 mg of frontal cortex tissue using RNeasy Mini Kit (Qiagen, cat no. 74104) according to manufacturer’s instructions. Final RNA was eluted using 30 μl of RNase, DNase free water and the concentration was measured with Nanodrop spectrometer. cDNA was synthesized, per mouse sample, with 20 ng of input RNA using the QuantiTect Reverse Transcription kit (Qiagen, cat no. 205311). Specific gene primers (Qiagen, cat no. 249900) were plated into 96 well qPCR plate with appropriate amount of cDNA synthesis product and QuantiTect SYBR green Master mix (Qiagen, cat no. 204143). Plates were sealed with Bio-Rad Micro Seals (Bio-Rad, cat no. MSB1001) and spun at 300 g for 30 s. All qPCR reactions were performed using Bio-Rad CFX96 Real-Time system C1000 Touch Thermocycler (settings: 95 °C for 15 min and 40 cycles of 94 °C for 15 s, 56 °C for 30 s and 72 °C for 30 s). Mouse QuantiTect assay primers were used to detect the following genes: *Actb* (Qiagen, cat no. QT00095242), *Hprt* (cat no. QT00166768), *Gapdh* (cat no. QT00199388), *Vcam1* (cat no. QT00128793), *Icam1* (cat no. QT00155078), *Icam2* (cat no. QT00097041) and *Serpine1* (cat no. QT00154756).

### Total tau ELISA

Total human tau levels were detected using electrochemiluminescence assay, MSD MULTI-Spot phospho (Thr231)/ Total tau Assay (Meso Scale Discovery, K15121D) following manufacturer’s instructions. All protein samples were initially diluted to 1 mg/ml. Further dilutions were performed using 10% blocker A (MSD, R93BA-4) in 1X Tris wash buffer (R61Tx-2) as diluent. All blood vessel samples (human and mouse) were diluted to 1:50 from 1 mg/ml stock protein and all frontal cortex samples (human and mouse) were diluted to 1:1000 from 1 mg/ml stock protein. Twenty-five μl of diluted samples were added to each well. The plate was read using MESO QuickPlex SQ120 (Meso Scale Discovery). Tau concentration per sample were calculated using the calibration curve.

### Statistics

A Shapiro-Wilks normality test was used on all datasets with *n* > 5 and use of non-parametric tests are indicated in-text. Student’s t tests or Mann-Whitney U tests were used for comparisons of two groups. Two-way ANOVA investigating genotype and sex did not reveal any significant impact of sex on any measure included in these studies. To compare doxycycline treated and untreated groups, two-way ANOVA was used followed by Sidak’s multiple comparisons test. Repeated measures ANOVA followed by Sidak’s multiple comparisons test was used when investigating differences between brain and blood vessel bioactivity and tau measures in mice. Significant *p*-values of < 0.05 are reported. All data were plotted and analyzed using GraphPad Prism version 7.00 for Windows (GraphPad Software).

## Results

### Tau is closely associated with vasculature in humans and mice

**Fig. 1:**
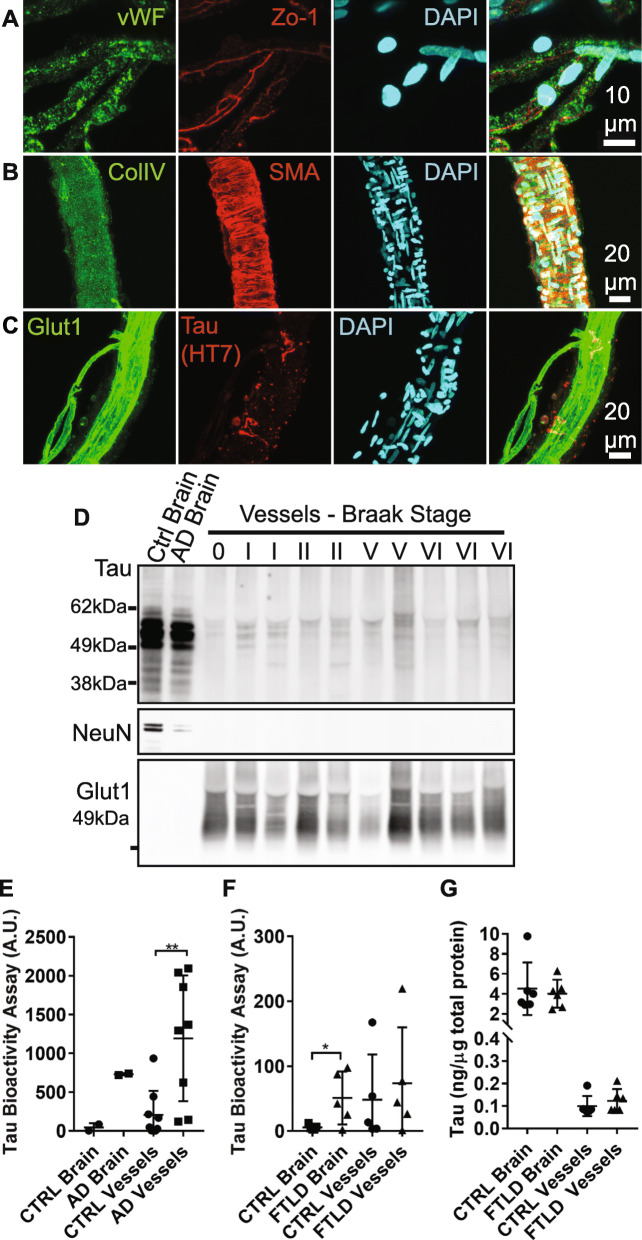
Tau protein in human vasculature. **a** Blood vessel isolations containing capillaries were validated by immunofluorescent labeling. DAPI-positive nuclei were also positive for endothelial cell markers such as von Willebrand factor (vWF) and zona occludins (ZO-1). **b** Isolates also contained basement membrane (collagen IV, ColIV) and some vessels were arteriolar in origin, indicated by the presence of smooth muscle actin (SMA). **c** Glut1 labeled endothelial cells were also occasionally observed to be surrounded by tau (HT7 antibody). **d** Western blotting of blood vessel isolations from human temporal cortex samples with varying degrees of tau pathology (Braak stage) indicates that vessels are frequently positive for tau (DAKO), but not other neuronal components (NeuN). Vessels are also enriched in Glut1 compared to total brain extracts. **e** When applied to a tau biosensor cell assay, vessels appear to retain their bioactivity with AD vessels exhibiting elevated seeding potential compared to controls (Mann-Whitney U test, *p* = 0.007, A.U. = arbitrary units). **f** Total frontal cortex protein from subjects with frontotemporal lobar dementia (FTLD) with tau exhibited greater bioactivity than control brains (Student’s t test, *p* = 0.038). **g** Total tau protein in total brain versus blood vessel protein preparations as measured by ELISA. All graphs are plotted with means +/− standard deviations. * indicates *p* < 0.05, ** *p* < 0.01

We hypothesized that tau may directly be contributing to the downstream vascular phenotype previously described in rTg4510 tau expressing mice [8]. Tau has been observed to be closely associated with vasculature in histology [18, 44, 53, 72]. To further explore these observations, we first isolated blood vessels from frozen human postmortem temporal cortex (Alzheimer’s) or frontal cortex (frontotemporal lobar dementia). In this modified protocol from Boulay et al. [14], tissue is first dounce homogenized, cell debris and myelin are removed using a dextran solution, and vessels are captured using nylon filters. We immunolabeled the resulting vascular preparations to confirm their purity, and observed classic endothelial cell markers such as von Willebrand factor (Fig. 1a, vWF), junction proteins such as zona-occludins (ZO-1), in addition to basement membrane (Fig. 1b, collagen IV) and smooth muscle cells (smooth muscle actin, SMA). Vessels purified by this method appeared to range from ~ 5–40 μm in diameter and primarily consist of capillaries. Importantly, vessels isolated from Alzheimer’s brain were occasionally observed to have tau labeling in close proximity to endothelial cells (Fig. 1c).

Immunoblotting of blood vessel isolates indicated that vessels are considerably enriched for vascular protein (Glut1) and not neuronal markers (NeuN), and that tau is common in these samples (Fig. 1d). A sensitive FRET-based HEK cell assay that measures bioactive tau, which are soluble tau species capable of inducing tau aggregation and are believed to contribute to the pathological spread of tau in the brain [31, 66], indicated bioactive tau is enriched in vessel preparations from Alzheimer’s disease (Fig. 1e) and appears to be elevated in frontotemporal lobar dementia-tau (FTLD-tau, Fig. 1f) and some control brains (Braak stages ≤ II). Although tau bioactivity was readily detectable, ELISA measurements of tau protein in vasculature were low and did not differ between Control and FTLD-tau samples (Fig. 1g, 0.12 +/− 0.05 ng tau per μg total protein in FTLD-tau vessels versus 4.0 +/− 1.4 ng tau per μg total protein in FTLD-tau cortex), suggesting that bioactive tau is enriched in association with microvasculature.

**Fig. 2:**
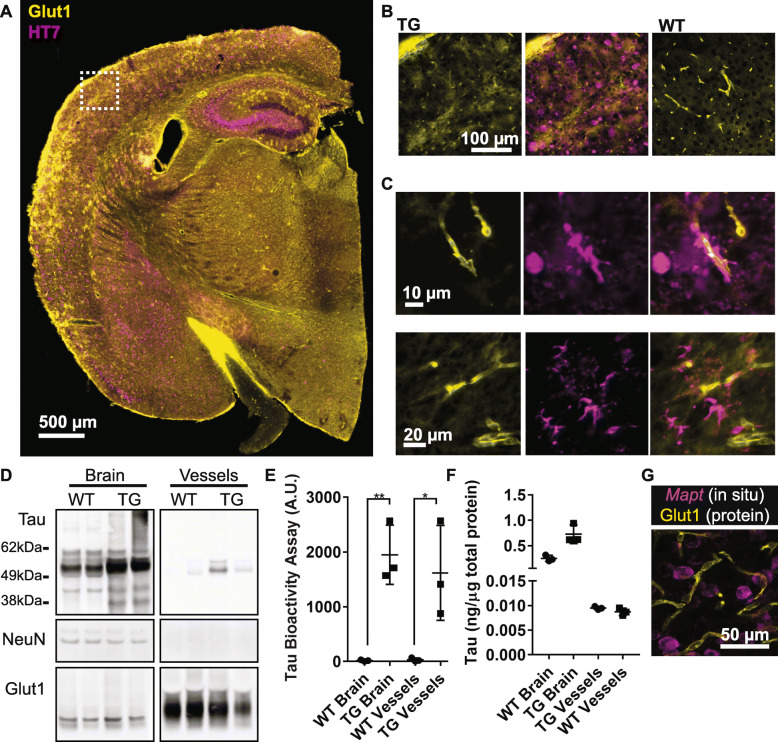
Tau protein in vasculature from transgenic mice. **a** Tissue sections from rTg4510 or wild-type controls at 15 months of age were labeled for tau (HT7) and endothelial cells (Glut1). **b** An enlarged view of the box from (**a**) shows the extent of tau pathology at this age in transgenic (TG) and not wild-type (WT) mice. **c** Numerous blood vessels were observed to be closely associated with tau. **d** Western blotting of total cortex and isolated vessels indicates tau is present in vessels, but not other neuronal components such as NeuN. Glut1 is highly enriched. Vessels and cortex protein were run on the same blot, but separated here for clarity. **e** Tau bioactivity in a biosensor cell assay shows increased tau bioactivity in transgenic brain versus wild-type (Repeated Measures ANOVA, Sidak’s post hoc, *p* = 0.003) and in transgenic blood vessels versus wild-type (Sidak’s post hoc, *p* = 0.01). **f** Tau protein ELISA measures in total brain versus blood vessel protein. **g** Expression of the human *Mapt* transgene was observed by in situ hybridization in neurons but did not co-localize with Glut1-positive endothelial cells. Image is of a single plane captured using a confocal microscope. All graphs are plotted with means +/− standard deviations. * indicates *p* < 0.05, ** *p* < 0.01

We also examined whether a comparable association of bioactive tau occurred in vessels in rTg4510 mice. Immunolabeling of rTg4510 or wild-type mouse brain showed that tau was observed in close proximity to vasculature, often appearing to encircle Glut1-positive endothelial cells (Fig. 2a-c). Tau was also detected in rTg4510 blood vessels by Western blot and total human tau ELISA (Fig. 2d, f) and tau present in vascular preparations retained bioactivity (Fig. 2e). Whole blood collected from a rTg4510 and prepared identically to vascular protein was not observed to possess any bioactivity in this measure (Tau bioactivity A.U. = 0.57 +/− 0.05, across three technical replicates), indicating residual blood in isolated vessels does not induce biosensor aggregation. As an additional control, in situ hybridization confirmed that endothelial cells do not express the tau transgene, (Fig. 2g). Together, these data confirm that bioactive tau species are present in the vascular bed and indicate tau may be directly responsible for some of the effects previously observed in rTg4510 mice.

### Suppressing tau expression is not sufficient to impact vascular remodeling

**Fig. 3:**
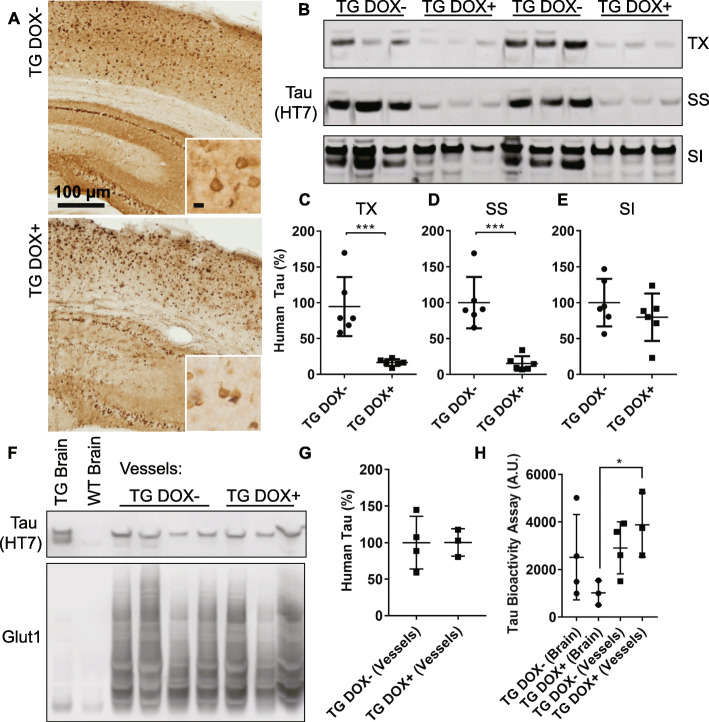
Doxycycline treatment reduces soluble tau but does not alter vascular tau. **a** Sections from rTg4510 mice (TG) with and without doxycycline treatment were labeled for tau protein (HT7) showing a significant reduction in tau outside of neurofibrillary tangles. Insets show higher magnification images of cortical pathology. Scale bar = 10 μm. **b** Western blotting of human tau (HT7) present in Triton X (TX), sarkosyl soluble (SS) and sarkosyl insoluble (SI) fractions from a protein insolubility assay. **c** Quantification of western blots confirms reduced soluble tau present in TX (Student’s t test, *p* = 0.001) and (**d**) SS fractions (*p* = 0.0002) and (**e**) no change in SI fractions (*p* = 0.31). Tau measures were normalized to a total protein stain to control for loading differences, which can be found along with uncropped blots in the supplement. **f** Western blotting of human tau in brain and isolated brain vessels from doxycycline treated (+) and untreated (−) mice. Glut1 is included to show enrichment of endothelial protein in vessels preparations. **g** Quantification of total human tau in vessels normalized to Glut1. **h** A biosensor cell assay shows retained tau bioactivity in transgenic blood vessels from Dox + mice (Repeated Measures ANOVA, Sidak’s post hoc *p* = 0.04) and no difference in Dox- mice (*p* = 0.89). All graphs are plotted with means +/− standard deviations. * indicates *p* < 0.05, ***p < 0.001

In the rTg4510 model, tau is expressed under a tetracycline-repressible promoter and feeding mice a diet containing doxycycline can halt the progression of tau pathology and neurodegeneration [61]. Due to the previously described increase in small diameter capillaries at 15 months, but not at 12 months of age in these mice [8], we fed mice doxycycline from 12 to 15 months of age. Turning off tau expression at this age appeared to eliminate tau in the surrounding neuropil/parenchyma, leaving only neurofibrillary tangles (Fig. 3a). Total tau burden was reduced by nearly half as measured by western blotting (Supplemental Figure 1A, B, 45% +/− 11% Dox + versus Dox-). Further biochemical fractionation confirmed that soluble tau species were significantly reduced (Supplemental Figure 2, Fig. 3b-d; Triton X, 16.4% +/− 4.7% Dox + versus Dox-; Sarkosyl soluble, 15.3% +/− 10% Dox + versus Dox-) but not sarkosyl insoluble tau (Fig. 3b, e; 79.7% +/− 33% Dox + versus Dox-). Levels of NeuN, an indicator of neurons, were unaltered between transgenic mice with and without doxycycline treatment (Supplemental Figure 1A, C).

To investigate changes in vascular associated tau, we isolated blood vessels from rTg4510 and wild-type control mice. By western blot, control mice were also observed to have endogenous tau associated with blood vessels (Supplemental Figure 3). Tau reduction did not alter the total amount of human tau associated with blood vessel isolates (Fig. 3f, g) and tau bioactivity was retained in isolated vessels from doxycycline treated mice versus whole brain (Fig. 3h).

**Fig. 4:**
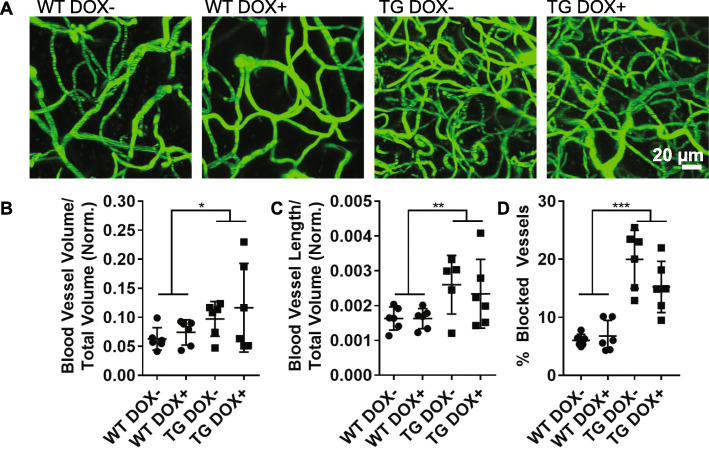
Vascular remodeling is not altered in mice with reduced tau burden. **a** 100 μm thick z-projection images of fluorescein-labeled vasculature from wild-type (WT) and transgenic (TG) mice captured by two-photon microscopy. **b** The volume of blood vessels in each mouse was calculated and normalized to the cortical thickness to account for atrophy. Blood vessel volume was increased in transgenic mice and was not altered by treatment (Two-way ANOVA, genotype *p* = 0.04, treatment *p* = 0.4). **c** Total blood vessel length was also increased in transgenics (Two-way ANOVA, genotype *p* = 0.007, treatment *p* = 0.74). **d** The total percentage of all vessels imaged without red blood cell flow was also increased (Two-way ANOVA, genotype *p* < 0.0001, treatment *p* = 0.19). All graphs are plotted with means +/− standard deviations. * indicates *p* < 0.05, ** *p* < 0.01, ***p < 0.001

To assess vascular remodeling in mice with reduced cortical tau burden, two-photon imaging of brain vasculature revealed an increase in total blood vessel volume (Fig. 4a, b) and length (Fig. 4c) in rTg4510 mice with and without doxycycline. In accord with this, we also observed that rTg4510 mice had a greater number of vessels devoid of red blood cell flow compared to wild-type, and suppressing tau expression did not affect this (Fig. 4d).

**Fig. 5:**
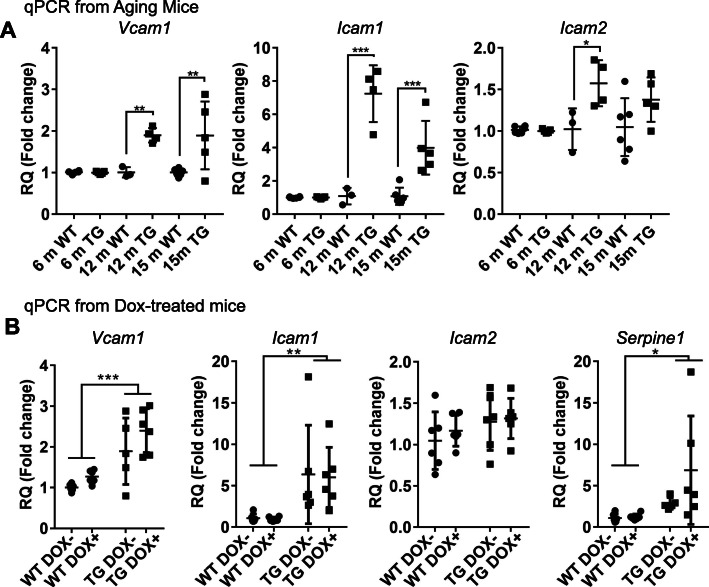
Increased cell adhesion protein expression in tau expressing mice. **a** Quantitative PCR revealed an increase in *Vcam1* (Two-way ANOVA, age *p* = 0.0117, genotype *p* = 0.0002, interaction p = 0.01), *Icam1* (age *p* < 0.0001, genotype *p* < 0.0001, interaction *p* < 0.0001), and *Icam2* (age *p* < 0.04, genotype *p* < 0.004, interaction *p* = 0.05). Increased expression was observed in transgenic 12-month mice and 15-month mice; Sidak’s multiple comparisons *p*-values are indicated. **b** Doxycycline treated and untreated transgenic mice had elevated *Vcam1* (Two-way ANOVA, genotype *p* < 0.0001, treatment *p* = 0.06, interaction *p* = 0.56), *Icam1* (genotype *p* = 0.002, treatment *p* = 0.90, interaction *p* = 0.94) and *Serpine1* (genotype *p* = 0.02, treatment *p* = 0.16, interaction *p* = 0.19), but not *Icam2* (genotype *p* = 0.13, treatment *p* = 0.50, interaction *p* = 0.74). Untreated 15-month mice are the same in panels (**a**) and (**b**). RQ = relative quatification using comparative Ct method. All graphs are plotted with means +/− standard deviations. * indicates *p* < 0.05, ** *p* < 0.01

Considering the increased proportion of capillaries without normal blood flow in rTg4510 mice, which we previously observed to be due to leukocyte adhesion to the endothelium [8], we investigated whether increased expression of cell adhesion molecules (CAMs) could be contributing to this phenotype. In rTg4510 mice, *Vcam1, Icam1*, and *Icam2* were all observed to be upregulated in the brains of 12-month-old mice with increased *Vcam1* and *Icam1* expression persisting at 15 months in transgenic mice (Fig. 5a). No differences between control and rTg4510 mice were observed at 6 months of age. Suppressing tau expression did not affect the increase in cell adhesion molecules (Fig. 5b). Similarly, elevation of *Serpine1,* a gene previously shown to be elevated in vasculature remodeling [8], was not affected by doxycycline treatment. Thus, despite widespread suppression of soluble tau, vascular-associated tau is retained and vascular remodeling and related inflammation-associated gene expression changes persist.

### Blood brain barrier (BBB) compromise is not reversible in aged mice

**Fig. 6:**
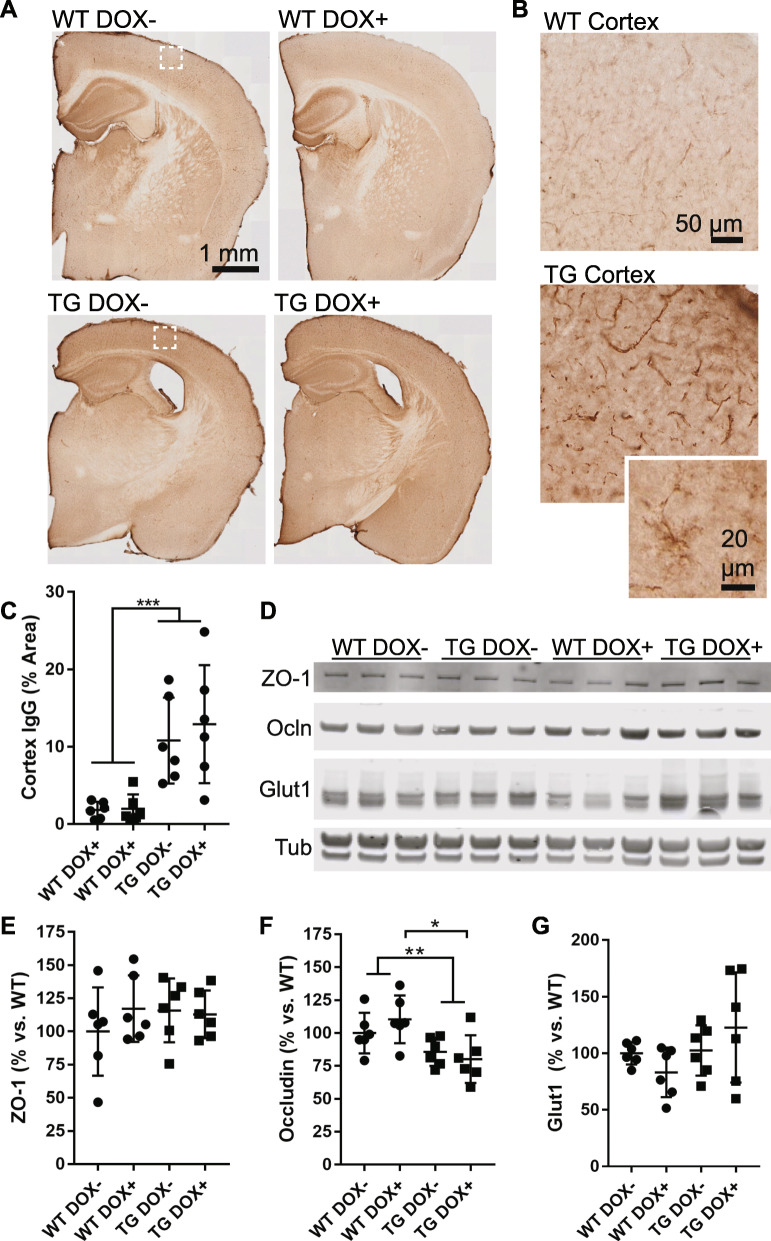
Compromised blood brain barrier function is not restored in tau expressing mice. **a** Sections from wild-type (WT) and transgenic (TG) mice were labeled to detect mouse IgG, which is normally excluded from the brain or found only within vascular lumens. **b** Enlarged views of boxes from (**a**) show that mouse IgG was detected in both wild-type and transgenic cortex, but to a greater extent in transgenic mice, including within vascular walls and in cells with glial morphology (inset). **c** A threshold-based quantification of cortical IgG labeling shows an increased in transgenic mice (Two-way ANOVA, genotype *p* < 0.0001, treatment *p* = 0.06). **d** A Western blot of tight junction proteins ZO-1 and Occludin (Ocln) as well as Glut1 (endothelial cell marker) and tubulin (loading control). **e** Quantification of the western blot from (**d**) showing ZO-1 protein normalized to Glut1 amount. Results are shown as the percentage versus wild-type (Dox-). No difference was seen in ZO-1 (Two-way ANOVA, genotype *p* = 0.59, treatment *p* = 0.50). **f** Quantification of Occludin normalized to Glut1 revealed a decrease in transgenic mice (Two-way ANOVA, genotype *p* = 0.003, treatment *p* = 0.71) including a significant difference between WT DOX+ and TG DOX+ (Sidak’s multiple comparison, *p* = 0.02). **g** Total vascular protein loaded on the Western blot was not significantly different between groups and is plotted normalized to tubulin (Two-way ANOVA, genotype *p* = 0.09, treatment *p* = 0.89)

A previous report has detailed vascular compromise in the rTg4510 mouse model is evident at 9 months and can be prevented by doxycycline suppression of the tau transgene [12]. Given the vascular changes that we observe at 15 months, we assessed blood brain barrier leakage by IgG immunostaining (Fig. 6a-c) and saw that both wild-type and rTg4510 mice had IgG labeling within the walls of blood vessels at this age but that the amount of this labeling was increased in rTg4510 mice. Sparse labeling of glia indicates extravasation and uptake into cells near leaky vessels [58]. Similar to other vascular phenotypes, doxycycline treatment and tau suppression from 12 to 15 months of age did not reduce the extent of IgG labeling that was observed in the cortex of rTg4510 mice.

To further explore changes contributing to the increased BBB leakage in rTg4510 mice, we performed western blotting for the tight junction proteins ZO-1 and Occludin and normalized to the total amount of Glut1 (endothelial cells; Fig. 6d-g). No difference was observed in the total amount of ZO-1, but Occludin was reduced in rTg4510 mice and was not improved with doxycycline treatment. These data indicate that vascular barrier functions continue to be impaired at this later age, even with significant reduction of soluble tau.

Of note, doxycycline has been reported to specifically reduce both protein and transcript levels of matrix metalloproteinase 9, which is capable of degrading tight junction proteins including Occludin [29, 73, 77]. To examine whether this effect of doxycycline per se was relevant using the mouse model and doses examined in our study, qPCR of *Mmp-9* in the brains of treated and untreated mice was performed. In these mice, treatment did not reveal a significant difference in any group (Two-way ANOVA, genotype *p* = 0.66, treatment *p* = 0.36).

## Discussion

From these data, it is apparent that tau protein is closely associated with vasculature in both human and mouse brain and that reduction of soluble tau burden, dramatically reducing tau outside of neuronal cell bodies, is not sufficient to reduce vascular tau and prevent microangiopathy in this mouse model. This indicates that microangiopathy, including increased numbers of small diameter capillaries, increased capillaries without normal blood flow, and increased BBB leakiness may be irreversibly set in motion at 12 months of age.

Analogous changes to vasculature have been reported in other diseases, possibly shedding light on the underlying cause of microangiopathy. In particular, increased expression of cell adhesion molecules such as VCAM1, ICAM1, and ICAM2 is a feature of proliferative diabetic retinopathy (PDR), where they increase recruitment of leukocytes to the endothelium and disrupt blood flow, a process termed leukostasis [19]. In PDR, high glucose levels in blood leads to inflammatory gene expression in endothelial cells including upregulation of CAMs [49]. Upon restoration of normal glucose metabolism, worsening of retinopathy can occur with persistent inflammation in the endothelium, disrupted blood flow, and sprouting of new, often leaky, vessels [5, 19].

Here, we see similar changes: even after tau production is suppressed, upregulated CAM expression and blood brain barrier leakage persists (although see below regarding the relatively long residence time of tau associated with vasculature in these experiments). In mice, we cannot completely rule out the possible contribution of genetic disruptions due to transgene insertions [26]. However, all experiments were conducted by comparing to littermate controls that possess the same genetic disruptions but do not express the tau transgene, indicating that these changes are likely due to tau and tau-driven inflammatory processes. In addition, these changes appear to reflect human disease; for example, increased soluble VCAM has been detected in the cerebrospinal fluid and plasma of Alzheimer’s disease subjects [37, 80]. Similarly, ultrastructural studies have provided evidence of altered blood brain barrier properties in Alzheimer’s [65] which have also been observed using contrast MRI reveal changes in blood brain barrier function [47, 52]. Altered expression of tight junction proteins have also been reported in Alzheimer’s disease, notably including reduced Occludin with increasing tau accumulation [76], which was also observed in this mouse model.

Considering the close association that we observed of bioactive tau and blood vessels, and its retention within the vascular compartment despite overall tau reduction, we also cannot rule out a direct effect of tau on stimulating endothelial cell inflammation. In fact, recent evidence indicates LRP1, a receptor enriched in this cell type, is a major route of tau endocytosis in cells and contributes to the internalization of pathological tau species [46, 57]. We observed limited co-localization of tau in endothelial cells in our experiment, however, and conclude that internalization and aggregation of tau in endothelial cells is likely a rare event in this model. Further, transcriptomic studies of brain endothelial cells from mouse and human indicate that endothelial cells do not produce endogenous tau [30, 59, 70, 78, 79]. Thus, the tau protein we observed in the vascular compartment is most likely of neuronal origin, and enrichment of tau in both para- and perivascular spaces is probably the result of brain clearance pathways [4, 34, 35, 54]. Presence of non-pathological endogenous mouse tau in blood vessel isolates supports this. Whether or not tau may directly lead to inflammatory changes in endothelial cells or if inflammatory signaling from glia and neurons leads to these changes is an important future direction.

Other important future studies might assess the contribution of concurrent vascular pathologies including CAA and cerebrovascular disease to the changes observed here. While in these studies we specifically avoided selecting Alzheimer’s cases with CAA to reduce potential confounds due to the presence of amyloid beta, evidence from mice indicates that CAA inhibits brain vascular clearance pathways and thus might enhance accumulation of vascular tau [2, 71]. This could provide some explanation for the apparent synergistic effect between CAA and the development of pathological tau accumulation [39, 53, 72, 74].

Related to this, the presence of amyloid beta has been shown to increase bioactive tau [7, 27, 42, 55]. Tau isolated from vasculature of Alzheimer’s disease blood vessels appeared to possess greater seeding activity on the biosensor assay than FTLD-tau vessels, which performed similar to some controls. While this could be due to inherent differences between vessels in temporal versus frontal cortex and further assessment of regional variability is necessary, we propose that the low amyloid plaque load in these FTLD-tau brain could be one possible reason for these differences, though it is worth noting that FTLD-tau is a broad category encompassing multiple pathologies which may be more or less associated with vascular changes [67]. In general, our data comparing FTLD-tau and control tissue tau levels is in line with previous reports indicating total tau is less effected in FTLD-tau subjects than in Alzheimer’s disease [62]. Further, CSF-tau levels are not frequently elevated in FTLD-tau [9], possibly reflecting differentially affected brain clearance pathways in these diseases which might contribute to reduced bioactivity in vasculature as measured here.

These data have clear implications for treating tauopathies including Alzheimer’s disease. In particular, this work highlights the importance of not only considering the critical timing of tau reduction therapeutics, but also various time scales of tau elimination from different compartments. For example, although doxycycline treatment leads to reductions in soluble tau, the number of aggregated neurofibrillary tangles is unchanged [61]. In the current study, we find little reduction in tau levels in vascular compartments even after 3 months of genetic suppression, suggesting that tau measured either biochemically or by a bioactivity assay in this compartment is unusually stable. By comparison, transgene suppression in younger mice [61] and similarly aged mice [17], has been shown to reduce soluble tau levels and promote better neuronal and behavioral outcomes. Thus, either more extensive reduction in tau, or a substantially longer treatment, may be necessary to impact tau dependent vascular alterations.

Alternatively, novel therapies directed at alleviating vascular dysfunction alongside tau-reducing strategies may be beneficial. An exciting target as suggested by this work is inhibition of endothelial cell adhesion molecule upregulation, which we observed concurrent with decreased numbers of blood vessels exhibiting normal blood flow. It is unknown whether this is similarly the substrate for reduced blood flow observed by neuroimaging studies [25, 36, 75], though it suggests that therapeutics aimed at alleviating leukocyte-endothelial interactions may provide some benefit.

Finally, given the clear overlap between the pathways of vascular dysfunction observed in tau mice and AD with that of common co-morbid diseases such as diabetes, this suggests that reinforcing pathways might lead to cumulative or even synergistic microvascular changes in some patients. In following, specific patient subgroups would benefit from an approach targeted to microvascular dysfunction, and identifying specific biomarkers to identify these individuals should be a priority.

## Supplementary information

**Additional file 1: Supplementary Table 1.** Neuropathological summary of human tissues included in these studies. **Supplemental Figure 1.** Western blot of total human tau in brain protein extracts. **Supplemental Figure 2.** Uncropped Western blots (from Fig. 3). **Supplemental Figure 3.** Western blots of tau in isolated vasculature from littermate wild-type control and rTg4510 mice.

## Data Availability

All data generated or analyzed during this study are included in this article and its supplementary files.
