# Supplementary material for: Tau reduction in aged mice does not impact Microangiopathy
Source: Acta Neuropathol Commun. 2020 Aug 18;8:137. doi: 10.1186/s40478-020-01014-4 (PMC7436970; doi:10.1186/s40478-020-01014-4)
Supplement: Supplementary file 1 — Additional file 1: Supplementary Table 1. Neuropathological summary of human tissues included in these studies. Supplemental Figure 1. Western blot of total human tau in brain protein extracts. Supplemental Figure 2. Uncropped Western blots (from Fig. 3). Supplemental Figure 3. Western blots of tau in isolated vasculature from littermate wild-type control and rTg4510 mice. [file 40478_2020_1014_MOESM1_ESM.docx]

| **Sample ID** | **ADNC A** | **ADNC B** | **ADNC C** | **Age at Death** | **Sex** | **Thal Stage** | **Braak Stage** | **PMI** | **NPDX** | **Vasc.**  **Path.** | **FC** | **TC** |
| --- | --- | --- | --- | --- | --- | --- | --- | --- | --- | --- | --- | --- |
| AD1 | 2 | 3 | 2 | 80 | M | 3 | VI | 12 | ADNC |  |  | x |
| AD2 | 3 | 3 | 3 | 64 | F | 4 | VI | 38 | ADNC |  |  | x |
| AD3 | 2 | 3 | 2 | 87 | M | 3 | VI | 24 | ADNC |  |  | x |
| AD4 | 3 | 3 | 1 | 90+ | F | 4 | V | 4 | ADNC |  |  | x |
| AD5 | 2 | 3 | 2 | 84 | F | 3 | VI | 10 | ADNC |  |  | x |
| AD6 | 2 | 3 | 2 | 89 | M | 3 | V | 16 | ADNC | CVD |  | x |
| AD7 | 2 | 3 | 2 | 89 | M | 3 | VI | 24 | ADNC |  |  | x |
| AD8 | 3 | 3 | 3 | 73 | F | 5 | VI | 22 | ADNC | CVD |  | x |
| AD9 | 2 | 3 | 2 | 90+ | M | 3 | VI | 12 | ADNC | CVD |  | x |
| CTRL1 | 0 | 0 | 0 | 86 | M | 0 | 0 | 10 | Control |  | x | x |
| CTRL2 | 0 | 1 | 1 | 90+ | M | 0 | I | 24 | Control |  | x | x |
| CTRL3 | 0 | 1 | 1 | 90+ | M | 0 | II | 12 | Control | CVD |  | x |
| CTRL4 | 2 | 1 | 1 | 90+ | M | 3 | II | 23 | Control | CVD |  | x |
| CTRL5 | 0 | 0 | 0 | 58 | F | 0 | 0 | 18 | Control |  |  | x |
| CTRL6 | 1 | 0 | 0 | 54 | M | 2 | 0 | 6 | Control |  |  | x |
| CTRL7 | 1 | 1 | 1 | 76 | F | 2 | I | 39 | Control | CAA |  | x |
| CTRL8 | 0 | 1 | 0 | 90+ | F | 0 | II | 24 | Control |  | x | x |
| CTRL9 | 0 | 1 | 0 | 90+ | M | 0 | II | 21 | Control | INF | x |  |
| CTRL10 | 1 | 1 | 1 | 90+ | F | 1 | I | 8 | Control |  | x |  |
| CTRL12 | 0 | 1 | 0 | 78 | F | 0 | I | 6 | DLDH |  |  | x |
| FTLD1 |  |  |  | 70 | M |  |  | 2 | FTLD-tau |  | x |  |
| FTLD2 |  |  |  | 90+ | F |  |  | NA | FTLD-tau |  | x |  |
| FTLD3 |  |  |  | 71 | F |  |  | 12 | FTLD-tau |  | x |  |
| FTLD4 | 1 |  | 0 | 56 | M | 1 |  | NA | FTLD-tau | CVD | x |  |
| FTLD5 | 1 | 2 | 1 | 71 | F | 2 | IV | 4 | FTLD-tau |  | x |  |

**Supplementary Table 1:** Neuropathological summary of human tissues included in these studies. NIA-AA criteria were used to rate pathology using the ABC scoring system. FC and TC columns indicate (‘x’) whether tissue was collected from frontal cortex BA 9 or temporal cortex BA 22 respectively. All control subjects were cognitively normal with the exception of CTRL12, which had no notable neuropathology on examination (DLDH = dementia lacking distinctive histology). FTLD 4 and 5 subjects were known *MAPT* P301L mutation carriers. Presence of cerebrovascular pathology is noted under a separate column (Vasc. Path.). Alzheimer’s Disease Neuropathological Change (ADNC), post-mortem interval (PMI), neuropathological diagnosis (NPDX), cerebrovascular disease (CVD), cerebral amyloid angiopathy (CAA), infarcts (INF).


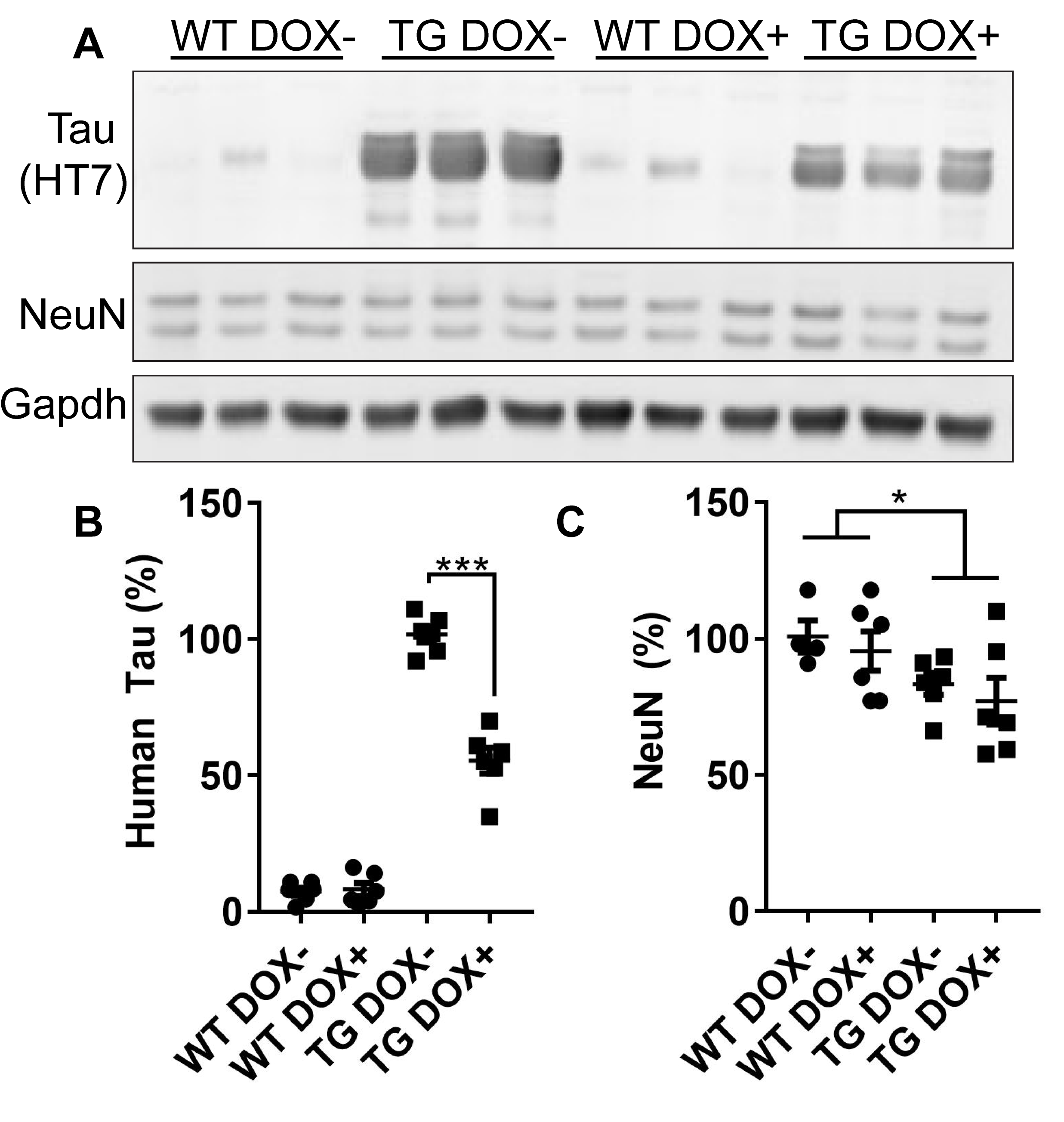


**Supplemental Figure 1: (A)** Western blotting of total human tau in brain protein extracts confirms histology findings. **(B)** Quantification of western blot from (B) indicates a significant reduction in tau protein in transgenic mice (Student’s t test, p<0.0001). **(C)** A significant difference in the amount of NeuN was observed between genotypes but not between treatment groups (Two-way ANOVA, genotype p=0.02, treatment p=0.42). All graphs are plotted with means +/- standard deviations. * indicates p<0.05, ***p<0.001.


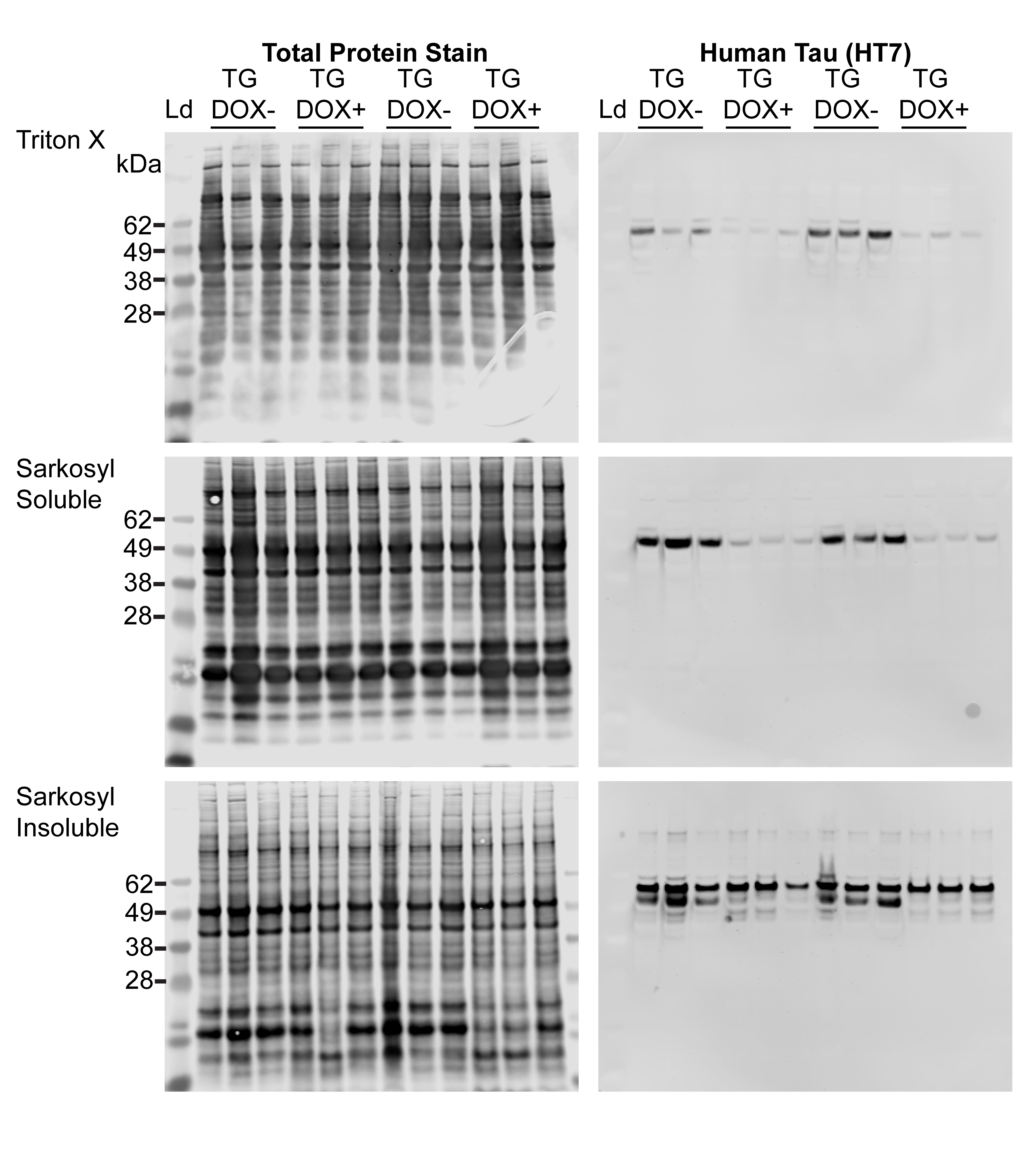


**Supplemental Figure 2:** Uncropped Western blots (from Figure 3) showing total protein stain used to as a loading control for normalization of total human tau probed with monoclonal tau HT7 antibody. Ladder (Ld) = Seeblue Plus2 Protein Standard with molecular weights labeled.


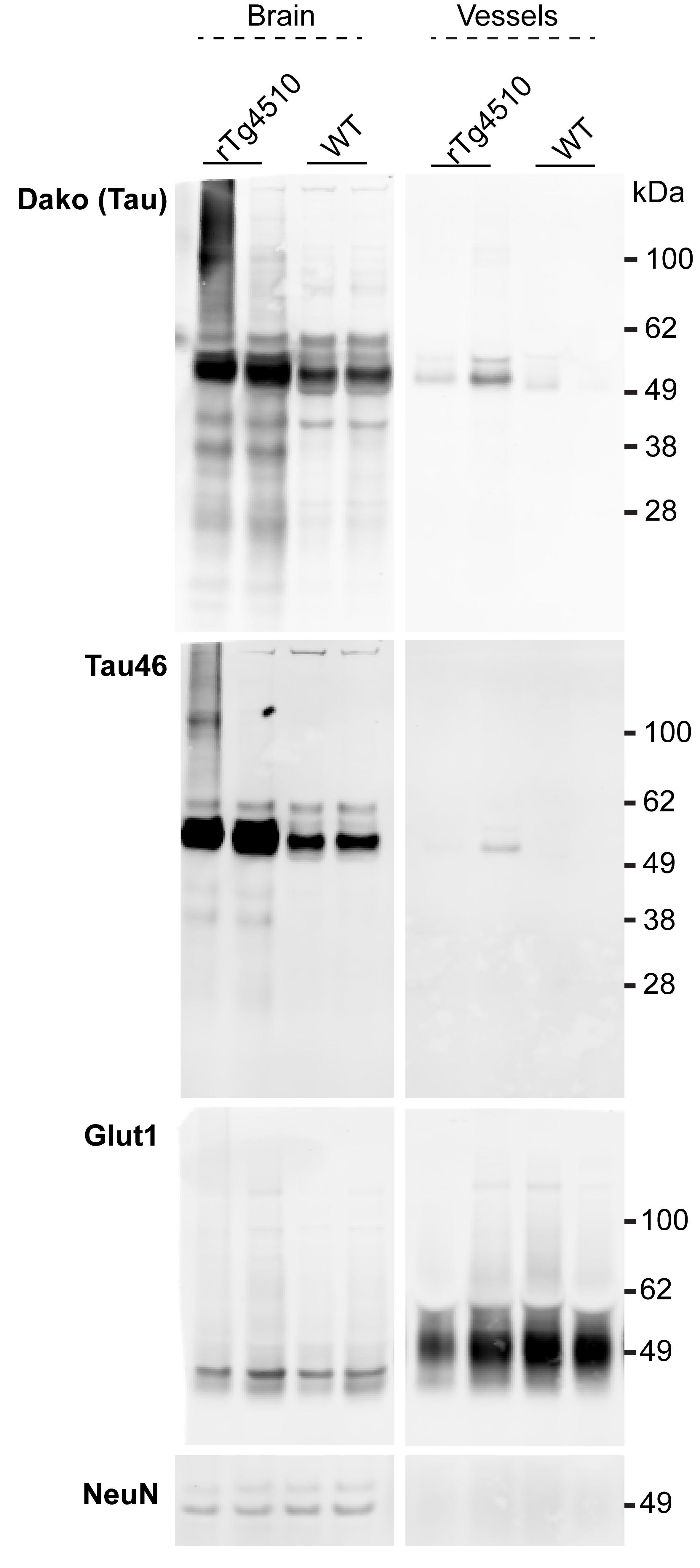


**Supplemental Figure 3:** Vasculature was isolated from littermate wild-type control and rTg4510 mice and blotted with a polyclonal tau antibody that detects both human and mouse tau (DAKO) or a monoclonal tau antibody that recognizes the C-terminus of both human and mouse tau (Tau46). Vascular preparations were enriched in endothelial cell Glut1 but not neuronal NeuN. Brain and blood vessel proteins were run on the same blot but edited for presentation.
